# Fish consumption is associated with school performance in children in a non-linear way: Results from the German cohort study KiGGS

**DOI:** 10.1093/emph/eoz038

**Published:** 2019-12-23

**Authors:** A Lehner, K Staub, L Aldakak, P Eppenberger, F Rühli, R D Martin, N Bender

**Affiliations:** 1 Institute of Evolutionary Medicine, University of Zurich, Winterthurerstrasse 190, 8057 Zurich, Switzerland; 2 Integrative Research Center, The Field Museum, 1400 S. Lake Shore Drive, Chicago, IL 60605-2496, USA

**Keywords:** fish, omega-3, cognition, long-chain fatty acids, brain evolution, brain development

## Abstract

**Introduction:**

How the long-chain fatty acids docosahexaenoic acid (DHA) and eicosapentaenoic acid (EPA) in the diet permitted human brain evolution, and how much our brains need today to function optimally are still hot topics for debate. DHA and EPA are considered as semi-essential because only insufficient amounts can be produced from other nutrients, such that they must be ingested with the diet. However, the Dietary Reference Intake of DHA and EPA, or of fish containing these fatty acids, has not yet been established. Eating fish is often recommended and generally considered beneficial for health and cognitive development in children and adolescents. For this study, data from a large cohort study were analysed to assess the association between fish consumption and cognitive school performance in children and adolescents.

**Methods:**

Data from the German cohort of children and adolescent health KiGGS, which was conducted 2003–06 and included more than 17 000 children, were analysed. Ordered logistic regressions were performed to test for associations between fish intake and school performance. Potential confounders were included in the models.

**Results:**

A statistically significant association was found between an intake of 8 g of fish per day and the probability of increasing the final grade in German [odds ratio (OR) 1.193, 95% confidence interval (CI) 1.049–1.358] and mathematics (OR 1.16, 95% CI 1.022–1.317) by one point, compared to no or very limited fish consumption. For the outcome German, higher levels of fish intake also showed a positive effect. These relationships were not linear but tended to decrease again at higher doses of fish.

**Discussion:**

Our result confirms previous reports of a positive association between fish intake and school performance. Interestingly, this relationship was not linear but tended to decrease again in the highest categories of fish intake. We hypothesize that mercury or other pollutants in the fish could be detrimental at high levels. As only half of all children met the minimal fish intake recommendations, fish consumption should be promoted more strongly in children to meet the minimal requirements of long-chain polyunsaturated fatty acids.

**Lay Summary:**

Polyunsaturated fatty acids like DHA and EPA that are present in fish are essential for a healthy human brain development. We found a U-shaped association between fish intake and school performance in children. We hypothesize that mercury or other pollutants in the fish could be detrimental at high intake levels.

## INTRODUCTION

Omega-3 long-chain polyunsaturated fatty acids (n-3 LC-PUFAs), and especially docosahexaenoic acid (DHA), are essential nutrients for learning capacity and behaviour. It has been shown that DHA accumulates in the cerebral cortex and hippocampus, areas of the brain that are associated with learning and memory. The direct association with learning and memory was first shown in an n-3 LC-PUFA depleted primate more than three decades ago [1, 2], but the amount of n-3 LC-PUFA needed for healthy development of the human brain and its function is still unclear [3]. Around 60% of the human brain (dry weight) consists of lipid compounds. Of the total lipid content of the brain, 35–40% are PUFAs, mainly the LC-PUFA eicosapentaenoic acid (EPA), DHA and arachidonic acid (AA). The brain’s fatty acid composition is conserved across many taxa. In all vertebrates, DHA is the major PUFA of the brain alongside AA [1, 4]. Large land-living mammals still conserve the same amounts of DHA in their brains as marine mammals, although levels of DHA and EPA are much lower in their food [1].

LC-PUFAs are divided into omega-6 LC-PUFA (n-6 LC-PUFA) and n-3 LC-PUFA. Both types are classified as essential fatty acids. Essential implies that they play a crucial role in human metabolism and that humans need to ingest them with their nutrition as they cannot be synthesized from other nutrients in sufficient quantities [5]. This incapacity exists because humans and other mammals lack two genes for the enzymes that incorporate a double bond at the omega-6 (Delta-12 desaturase absent) and omega-3 (Delta-15 desaturase absent) positions. As a consequence, linoleic acid (18:2omega 6) and alpha-linolenic acid (ALA) (18:3omega 3) have to be present in food and cannot be converted from the precursor oleic acid (18:1n-9) [6]. From this step onwards, the body can synthesize AA from linoleic acid and both EPA and DHA from ALA [5].

In plants, linoleic and ALA are found in substantial amounts in seeds and nuts with a considerable variation in fatty acid composition [5, 7]. Certain macro- and microalgae contain large amounts of DHA and EPA [7, 8]. Along the food chain, DHA and EPA spread first through the aquatic ecosystem and subsequently through the terrestrial ecosystem [9]. Besides algae, dietary sources that are richest in DHA and EPA are cold-water fish such as salmon, mackerel, halibut, sardines, tuna and herring [5, 7].

Recently, there has been a lively debate in the literature about how much DHA and EPA in the human diet was necessary for human brain evolution to take place, as well as about how much DHA and EPA is needed for optimal brain function in our diet today. The conversion rate from LC-PUFA to EPA and finally DHA can be greatly influenced by the n-6:n-3 ratio of the diet due to enzyme competition and by other factors like nutritional status and sex [10]. However, the conversion rates for EPA and DHA might have also changed during evolution as we know that conversion rates differ in sea animals, obligate land carnivores, humans and land-living herbivores, with the highest conversion rates in herbivores [11]. Such changes in conversion rates might influence estimates of the minimal necessary amounts of nutrients necessary for brain evolution if diet changed.

The Dietary Reference Intake of DHA and EPA has not yet been established, but it is expected that supplementation of the usual diet has a beneficial effect in the case of deficiency [12]. Systematic reviews have shown a benefit of n-3 LC-PUFA supplementation in the form of fish oil in cardiovascular and inflammatory diseases [13, 14]. Nutritional guidelines regularly recommend fish intake for adults, pregnant women and children to meet LC-PUFA requirements [15–17]. Furthermore, several cohort studies have confirmed the association between fish or PUFA intake and cognitive performance in children. A British cohort showed that children of mothers with a high fish intake during pregnancy achieved higher scores in the MacArthur Communicative Development Inventory at 15 months, as long as the fish was not contaminated with methylmercury [18]. Furthermore, frequent fish intake at age 15 showed a significantly higher cognitive performance 3 years later in a longitudinal cohort study in Swedish male adolescents [19]. Similar results were found in Chinese school children [20]. However, the exact amount and species of fish that meet the minimal physiological need are as yet unknown, especially for children of different ages. For this study, data from a large cohort study have been analysed to assess the association between fish consumption and school performance in children and adolescents.

## MATERIALS AND METHODS

The KiGGS study is a nationally representative cohort study conducted in Germany. Baseline data collection was conducted by the Robert Koch Institute (RKI) between May 2003 and May 2006. The gross sample size was around 32 400, and a total of 17 641 children and teenagers participated (participation rate of 66.6%), of which 8656 were girls and 8985 were boys. The sample points were 167 municipalities, including towns, representative for Germany [21, 22].

The aim of KiGGS was to obtain representative data about the health of children and teenagers from 0 to 17 years of age living in Germany. Special efforts were undertaken to include foreign children with language barriers in the survey as well. Children and teenagers living in medical facilities or care institutions were excluded. A total of seven questionnaires were used. For children 6–10 years of age, caregivers filled out the questionnaires for their children or together with them. For children and adolescents 11–17 years of age, one questionnaire was filled out by a parent, containing questions related to events further in the past and a second questionnaire assessing psychosocial well-being and health-related behaviour in teenagers was filled out by the children and adolescents themselves [21].

A broad spectrum of questions was used to retrospectively assess average nutrition using a ‘Food Frequency Questionnaire’ developed by the RKI. Questions about the frequency and amount of consumption were asked in the categories food, drinks and consumption of additional vitamins and minerals such as supplements and functional foods [23]. Around 50 food groups were covered in these questionnaires and portion size and information on frequency of consumption for each food group was sought.

In this study, the final grades achieved in German and mathematics were studied as proxies for cognitive performance, as no cognitive performance test was used in KiGGS. The German school system shows differences between regions but follows an overall standardized structure. Therefore, scholastic comparisons within the KiGGS study are possible [24]. The grades achieved were associated with fish intake and corrected for age, sex, Winkler Social Index, German language spoken at home, residence (rural or towns), school enrolment (earlier or later), school trajectory (skipped or repeated classes), body height, body weight and diagnosed attention deficit hyperactivity disorder (ADHS) cases. All variables were restricted to age 6 and above because children enter school at this age in Germany. The Winkler Social Index score is a quantitative summary of variables built by the RKI using the caregivers’ education, occupational position and the net income of all household members [25].

To situate fish consumption in relation to the healthiness of food intake in general, the consumption of other chosen food items that represent healthy (vegetables) and unhealthy (sweet beverages) nutrition were also considered. Meat consumption was assessed because it potentially competes with fish consumption. The mean frequency over 4 weeks was calculated for every food group (fish, meat, sweet drinks and vegetables). Subsequently, the mean daily amount (g) of each food group was calculated from portion size and frequency [25].

In Germany’s grading system, grade one is the highest and grade six is the lowest. To make the results more intuitive, we reversed this order for both mathematics and German grades, making six the highest grade. In the food and drink categories, the higher the category number the higher is the intake.

### Statistical analysis

Descriptive statistics were calculated for all variables considered, reporting numbers and percentages of children in each category of categorical variables (Table 1). Age was grouped into three categories (6–9, 10–13 and 14–17 years). Fish consumption was grouped into quintiles, and the corresponding mean amount of fish, number and percentage of children per category, and mean mathematics and German grade per category were reported (Table 2).

**Table 1.** Number and percentages of children per variable

	Number of children	Percentage of children	Data lacking
Age			0
6–9 years	4136	34.56	
10–13 years	4094	34.21	
14–17 years	3736	31.22	
Sex			0
Girls	5831	48.73	
Boys	6135	51.27	
Winkler Social Index			327
Low	3182	27.34	
Intermediate	5499	47.25	
High	2958	25.41	
Last mark mathematics			3346
1	13	0.15	
2	270	3.13	
3	1553	18.02	
4	3117	36.16	
5	2931	34	
6	736	8.54	
Last mark German			3361
1	4	0.05	
2	144	1.67	
3	1447	16.82	
4	3497	40.64	
5	2940	34.17	
6	573	6.66	
Speak German at home			205
Yes	11 327	96.31	
No	434	3.69	
ADHS			1131
Yes	625	5.77	
No	10 210	94.23	
Location			0
Rural	2611	21.82	
Small town	3140	26.24	
Middling town	3457	28.89	
City	2758	23.05	
School flow			1376
All classes regular	8830	83.38	
One or more classes ahead	84	0.79	
One or more classes repeated	1267	11.96	
Still in first class	409	3.86	
Sweet drinks			711
1 (8.64 g/day)	2302	20.45	
2 (27.47 g/day)	2804	24.91	
3 (45.88 g/day)	1780	15.82	
4 (66.99 g/day)	2309	20.52	
5 (138.68 g/day)	2060	18.3	
Vegetables			847
1 (18.94 g/day)	2225	20.01	
2 (53.17 g/day)	2223	19.99	
3 (92.39 g/day)	2346	21.1	
4 (144.45 g/day)	2143	19.27	
5 (296.39 g/day)	2182	19.62	

**Table 2.** Description of fish consumption categories

Fish category	Mean amount of fish consumption (g/day)	Number of children	Percentage of children	Mean grade mathematics	Mean grade German
1	0.47	2338	20.71	3.42	4.22
2	3.9	2525	22.37	3.39	4.24
3	8.04	2721	24.11	3.43	4.39
4	17.25	2871	25.43	3.34	4.33
5	52.24	833	7.38	3.47	4.16

Associations of fish intake with the final grade in mathematics and the final grade in German were assessed using ordered logistic regressions. Both models contained the mean daily amount of fish consumption as the main independent variable. The models were controlled for the mean daily amount of vegetables, mean daily amount of sweet drinks, mean daily amount of meat, age, sex, Winkler Social Index score, German spoken at home, ADHS, school enrolment, school flow, body height, body weight and location (rural vs cities of different sizes). As there were missing data for some variables, the total of children included in the regressions was reduced to those with a complete dataset. In a second analysis step, the variables that showed no significant association were removed from the final models. Stata© version 14.2, Stata, Corp., College Station, TX, USA, was used for all analyses.

## RESULTS

Because of the age restriction and missing data, a total of 8605–11 966 children were included in the descriptive analyses, and 7489–7495 children were included in the regression analyses. There were marginally fewer girls than boys, and most had an intermediate Winkler Social Index score. School grades had a similar distribution in mathematics and German, grade six (here the best) was given more than the lowest grade and most had grade four or five. More than 95% of the children spoke German at home. Almost 6% of all children were diagnosed with ADHS by a health professional. The children’s domicile was equally distributed between rural, small- and medium-sized towns and cities, with slightly more children from small- and medium-sized towns. Eighty-three percent of children passed through classes at the regular pace, exceptions mostly had to repeat one or several classes. The amount of sugar from sweetened drinks per day ranged from very low (9 g/day) to high intakes (139 g/day). We included body height as a continuous variable, with a mean of 150.52 ± 18.78 cm, and marginally left skewed. For details see Table 1.

The variable fish (mean daily amount of fish consumption in grams) was highly skewed towards a low fish intake, with a median of 8.04 g and a mean of 23.99 ± 11.15 g. We divided fish consumption into quintiles. The first category reflected a mean daily amount of 0.47 g and the fifth category 52.24 g. The fifth category contained the smallest number of children (833 observations). Mean grades in mathematics per fish category varied between 3 and 4; mean grades in German varied between 4 and 5. For details see Table 2.

**Figure 1.**
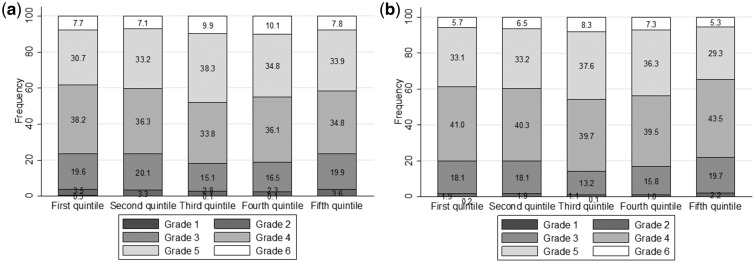
Bar chart displaying frequency of mathematics (**a**) and German (**b**) grades per fish category. The five categories correspond to the five quintiles of fish intake (1, lowest intake quintile; 5, highest intake quintile). The six grey shade codes correspond to the six grades (1, lowest grade; 6, highest grade)

Figure 1 shows the percentage of fish consumption per school grade, for mathematics and German separately. For both mathematics and German, the proportion of grades above 5 increases for categories 3 (∼8 g fish per day) and above. However, categories 4 and 5 again show lower proportions of high grades similar to categories 1 and 2. This means that, for mathematics as well as for German. The fish intake/grade relationship is inversely U-shaped.

The variables mean daily consumption of meat, body weight and time of school enrolment had no significant association with grades in mathematics or German. Consequently, these variables were removed from both models. Mean daily amount of vegetables showed an association with grades in German but not in mathematics. Therefore, this variable was kept in both models.

The final ordinal logistic regression model associating fish intake with the final grade in mathematics revealed a significant association with the third category of fish intake, which corresponds to a mean of 8.04 g of fish per day. This amount significantly increased the chance for one better grade point in mathematics by 16% (*P* < 0.05) compared to intake category 1 (no or almost no fish consumption). Consumption of more fish was, however, not associated with higher grades in mathematics. The same amount of fish intake (8.04 g/day) significantly increased the chances for a better grade in German by 19% compared to category 1, and the chances for a better grade in German were significantly increased by 16% and 24% for fish intake categories 4 and 5. Categories with a smaller amount of fish per day (categories 1 and 2) did not show a significant association with school performance.

The intake of vegetables increased the probability of achieving a higher grade in German between 15% and 25% in all but one category, while it had no association with the final grade in mathematics. The other variables showed significant associations in both models of school performance, with sweetened beverages decreasing school performance in a linear fashion (the more sweetened beverages consumed, the smaller the chances for a better grade). Younger children had better chances for better grades in both models. Boys showed a higher chance for better grades in mathematics, while girls showed a higher chance for better grades in German. Socioeconomic status is linearly associated with school performance, with higher status corresponding to higher probabilities of better grades. Living in rural areas increased the chances for better school performance compared to living in towns or cities. Interestingly, speaking German at home decreased the chances for better performance in mathematics, while it increased the chances for better performance in German. To suffer from ADHS decreased the chances for better school performance in both mathematics and German, and in both models children who were one or more classes ahead showed increased chances for better school performance, while children who had to repeat one or more classes showed decreased chances. Body height was negatively associated with school performance in both models (even after correcting for sex and age). Results are presented in Tables 3 and 4.

**Table 3.** Results of ordered logistic regression for the outcome final grade in mathematics (*n* = 7495)

Category	Odds ratio	95% confidence interval
Fish		
1	Baseline	
2	0.983	0.866–1.115
3	1.16^*^	1.022–1.317
4	1.055	0.93–1.983
5	1.125	0.937–1.352
Sweet drinks		
1	Baseline	
2	0.823^**^	0.722–0.938
3	0.709^***^	0.612–0.822
4	0.764^***^	0.666–0.876
5	0.632^***^	0.547–0.731
Vegetables		
1	Baseline	
2	1.048	0.915–1.201
3	1.059	0.926–1.211
4	1.146	0.998–1.316
5	1.053	0.917–1.209
Age		
1	Baseline	
2	0.451^***^	0.389–0.523
3	0.343^***^	0.279–0.42
Sex		
Boys	Baseline	
Girls	0.719^***^	0.658–0.786
Winkler Social Index		
Low	Baseline	
Intermediate	1.429^***^	1.285–1.59
High	2.241^***^	1.976–2.543
Location		
Rural	Baseline	
Small town	0.874^*^	0.777–0.983
Middling town	0.832^**^	0.74–0.935
City	0.875^*^	0.77–0.994
Speak German at home		
Yes	Baseline	
No	1.658^*^	1.137–2.417
ADHS		
Yes	Baseline	
No	2.165^***^	1.799–2.605
School flow		
Regular	Baseline	
One or more classes ahead	1.645	0.958–2.824
One or more classes repeated	0.412^***^	0.358–0.474
Still in first class	1.633	0.583–4.571
Body height	0.985^***^	0.98–0.99

* *P* < 0.05.

** *P* < 0.01.

*** *P* < 0.001.

**Table 4.** Results of ordered logistic regression for the outcome final grade in German (*n* = 7489)

Category	Odds ratio	95% confidence interval
Fish		
1	Baseline	
2	1.07	0.94–1.219
3	1.193^*^	1.049–1.358
4	1.158^*^	1.017–1.318
5	1.235^*^	1.026–1.487
Sweet drinks		
1	Baseline	
2	0.76^***^	0.664–0.868
3	0.629^***^	0.541–0.731
4	0.638^***^	0.555–0.733
5	0.587^***^	0.506–0.68
Vegetables		
1	Baseline	
2	1.152^*^	1.002–1.325
3	1.073	0.936–1.231
4	1.246^**^	1.082–1.435
5	1.243^**^	1.079–1.431
Age		
1	Baseline	
2	0.445^***^	0.382–0.518
3	0.39^***^	0.317–0.481
Sex		
Boys	Baseline	
Girls	2.113^***^	1.927–2.318
Winkler Social Index		
Low	Baseline	
Intermediate	1.632^***^	1.463–1.821
High	2.569^***^	2.257–2.924
Location		
Rural	Baseline	
Small town	0.898	0.796–1.013
Middling town	0.821^**^	0.728–0.925
City	0.904	0.794–1.029
Speak German at home		
Yes	Baseline	
No	0.59	0.404–0.864
ADHS		
Yes	Baseline	
No	2.313^***^	1.915–2.794
School flow		
Regular	Baseline	
One or more classes ahead	1.118	0.657–1.901
One or more classes repeated	0.392^***^	0.34–0.451
Still in first class	1.375	0.493–3.836
Body height	0.982^***^	0.978–0.987

* *P* < 0.05.

** *P* < 0.01.

*** *P* < 0.001.

## DISCUSSION

According to a nutritional survey in Germany (Nationale Verzehrstudie II), fish consumption in Germany is relatively low, especially in young people [26]. In this survey, a mean consumption of 5–6 g/day was found for juveniles, while a median consumption of 8 g/day (56 g/week) was found in KiGGS. From a mean daily amount of 8.04 g fish/day up to 52.42 g fish/day, the likelihood of a better grade in German was significantly increased by 19–24%. In mathematics, only the category with 8.04 g fish/day showed a significantly increased chance for a better grade. These results persisted after correcting for several significant co-variables such as sex, age, socioeconomic index, place of residence and ADHS diagnosis.

From a physiological point of view, these results are plausible: a minimum amount (threshold) may be required to avoid deficiencies and therefore show a positive effect. Once the body is sufficiently supplied, additional amounts will not lead to further improvement. Such a threshold seems apparent in both models, German and mathematics. The fact that in both models the same threshold was found (8.04 g/day) indicates that this amount might be close to the minimal requirement of fish-based nutrients for the human brain at the age of school children.

As far as we can judge, our results are consistent with those of similar observational studies. A British cohort showed that children of mothers with a higher fish intake during pregnancy achieved a higher score in the MacArthur Communicative Development Inventory at 15 months, as long as fish is not contaminated with methylmercury [18]. Frequent fish intake at age 15 showed a significantly higher cognitive performance 3 years later in a longitudinal cohort study in Swedish male adolescents [19]. Similar results were found with Chinese school children [20].

The fact that the outcome final grade in mathematics showed an improvement only in category 3 and the outcome final grade in German showed an effect for category 3 and all subsequent categories, needs further investigation. With both outcomes, higher categories tended to decrease again. A study in Dutch adolescents showed similar results [27]. In that study, fish intake was divided into more categories than usually used in similar studies, including a category with high intake. By dividing fish consumption into quintiles, we also created a category with high fish intake (category 5). In this way, both studies revealed a decreasing relationship between very high fish intake and school performance. This result could be explained by high mercury or other pollutant intake with high fish consumption, resulting in declining school performance.

The most consumed fish species in Germany in 2004 were Pollock (24.7%), herring (15.0%), tuna (12.6%), salmon (10.3%) and rose fish (5.8%). Among these species, the highest methylmercury content was measured in tuna (0.308 µg/g); in rose fish, there was a mean of 0.224 µg/g; in herring, 0.066 µg/g; and in salmon and Pollock, 0.028 µg/g [28]. The FAO/WHO Expert Committee on Food (JECFA) recommended in 2003 a maximum of 1.6 µg methylmercury per kg body weight as provisional tolerable weekly intake, and the National Research Council (NRC) of the US recommended an intake limit of 0.7 µg/kg body weight.

For an average adult person consuming 300 g fish per week, this results in 83% of the JECFA maximum and 189% of the NRC maximum for tuna, 60% and 137% for rose fish, 18% and 40% for herring and 8% and 17% for salmon and Pollock [29]. The KiGGS children ate 56 g per week on average, so even if they were smaller than adults, their mercury intake might have been lower than the figures given for adults. However, the children in the highest intake category (365 g fish per week) on average had a slightly higher fish intake than the mean amount assumed for adults. It cannot be excluded that some of these children could, therefore, have exceeded the methylmercury intake recommendations, especially if they ate more fish from the more polluted species such as tuna or rose fish.

Experts worldwide agree that eating fish is beneficial for children and adults. Besides the health-promoting omega-3 fatty acids (DHA and EPA), fish is an important source of high-quality protein and micronutrients. Protein, minerals and most vitamins are absent from DHA and EPA supplements [16]. Most experts agree that the nutritional benefits of eating fish outweigh the risk of methylmercury and persistent organic pollutants, but many recommend eating seafood with lower methylmercury levels and, in an ideal case, with a concurrent high DHA and EPA content [30]. Low-methylmercury seafood corresponds to animals from lower trophic levels, which tend to accumulate less contaminants. Clams and shrimps from the lowest trophic level contain <0.8 mcg/dl of mercury, while sharks from the highest trophic level contain up to 120.8 mcg/dl. Cold-water sourced salmon with its high DHA/EPA content and its particularly low mercury amount fulfils the requirements best [30].

For children and adults, the FDA recommends 2 and 3 servings of fish with the lowest mercury content and 1 serving of fish with medium mercury content per week. One serving for children between age 4 and 7 is 57 g (2 ounces) and for adults, it is 113 g (4 ounces) [16]. In Germany, the recommendation starts with 50 g/week for children at age 4 and goes up to 90 g/week for children of age 12. For adults 100 g/week of fish are recommended [17]. The guideline from the federal office of public health in Switzerland recommends 1 and 2 portions of fish per week for adults, which corresponds to 100–240 g of fish per week. Their recommendation for non-fish eaters is to ingest 500 mg DHA and EPA a day through fish oil supplementation [15]. Specific recommendations for juveniles and infants are lacking. In our analysis, category 3, which had a significant association with both school outcomes, reflects an average of 56.3 g fish/week, which corresponds to one portion for small children or half a portion for adults per week. The children in our analysis were between 6 and 17. This amount is therefore at the lower end of the German and American recommendation for fish consumption. Our results suggest that this amount might be sufficient for a beneficial effect on cognitive performance, and might minimize the detrimental effects of methylmercury. However, further studies will be needed to validate this assumption.

The DHA content varies significantly between fish species. An Atlantic salmon can contain 24 times more DHA than a shrimp [30]. Not only due to species, but also to diet, geographical origin, gender and season, the lipid content and composition of fish can vary. The total lipid content and the total n-3 LC-PUFA content of seawater species ranges from 2.6 times more up to 3.9 times more than in freshwater fish [31]. In seawater fish species, the highest n-6/n-3 ratio was found to be 0.59 (sea bass) and in freshwater it was 1.0 (North African catfish). In the KiGGS questionnaire, there was no differentiation between sea or freshwater fish species, climate zone, nor the kind of fish consumed. We are therefore unable to calculate intake for DHA or n-3/n-6 ratio from fish consumption.

A strength of our analysis is the large sample size of the KiGGS study. Another strength is the extensive array of potential confounders covered in the KiGGS study that we were able to include in our models. For instance, our models showed that many factors such as socioeconomic status, sex, age, place of residence or consumption of sweet beverages were strongly associated with school performance. By including these factors in the model, we could show an effect of fish consumption independent of these factors. The large sample size, the extensive correction for potential confounders, and the fact that both models pointed towards the same threshold for the minimal amount of fish per day required to improve cognitive performance, allow us to be confident that our findings are likely to be reliable.

A limitation of our analyses is that the questionnaire in the KiGGS study did not distinguish between fish species, so that LC-PUFA intakes can only be estimated. The questionnaire also did not identify any other consumed seafood besides fish. Another limitation is that the KiGGS study did not provide a specific cognitive test of the children, so that we had to adopt the final school grade in mathematics and in German as a proxy for cognitive performance. This measure is prone to bias due to many potential confounders for which we might not have corrected. As it might not be feasible during retrospective data collection to gather information on fish species or source (sea or freshwater, cold or warm climate), a good alternative data source would be to measure participants’ erythrocyte fatty acid status (total lipid content, total n-6 LC-PUFA, total n-3 LC-PUFA, DHA, EPA, AA) to determine whether DHA and EPA concentrations are associated with school performance [32].

According to the American or German Guidelines for fish consumption in childhood, the minimal daily fish intake is sufficiently met by half of the population in KiGGS. From these findings, we conclude that fish consumption should be promoted more strongly in children to meet the minimal requirements of LC-PUFAs in all children. If fish consumption is not possible due to allergy or other reasons, supplementation with fish oil or microalgal oil capsules should be considered.
